# The Anti-Inflammatory Effects of Broccoli (*Brassica oleracea* L. var. italica) Sprout Extract in RAW 264.7 Macrophages and a Lipopolysaccharide-Induced Liver Injury Model

**DOI:** 10.3390/cimb45110572

**Published:** 2023-11-14

**Authors:** Hyeon Woo Sim, Won-Yong Lee, Ran Lee, Seo Young Yang, Youn-Kyung Ham, Sung Don Lim, Hyun-Jung Park

**Affiliations:** 1 Department of Animal Biotechnology, College of Life Science, Sangji University, Wonju-si 26339, Republic of Korea; opensesim@gmail.com (H.W.S.);; 2 Department of Livestock, Korea National College of Agriculture and Fisheries, Jeonju-si 54874, Republic of Korea; leewy81@korea.kr; 3 Department of Biology Education, Teachers College and Institute for Phylogenomics and Evolution, Kyungpook National University, Daegu 41566, Republic of Korea; syy@knu.ac.kr; 4 Department of Animal Science, Sangji University, Wonju-si 26339, Republic of Korea; ykham21@sangji.ac.kr; 5 Department of Plant Life and Resource Science, Sangji University, Wonju-si 26339, Republic of Korea

**Keywords:** broccoli sprouts, anti-inflammation, cytokine, liver injury, lipopolysaccharide

## Abstract

*Brassica oleracea* var. italica (broccoli), a member of the cabbage family, is abundant with many nutrients, including vitamins, potassium, fiber, minerals, and phytochemicals. Consequently, it has been used as a functional food additive to reduce oxidative stress and inflammatory responses. In the current study, the effects of sulforaphane-rich broccoli sprout extract (BSE) on the inflammatory response were investigated in vitro and in vivo. Comparative high-performance liquid chromatography analysis of sulforaphane content from different extracts revealed that 70% ethanolic BSE contained more sulforaphane than the other extracts. qPCR and enzyme immunoassay analyses revealed that BSE markedly reduced the expression of proinflammatory cytokines and mediators, including cyclooxygenase 2, interleukin (IL)-1β, IL-6, IL-1, inducible nitric oxide synthase (iNOS), and tumor necrosis factor-α (TNF-α), in lipopolysaccharide (LPS)-stimulated RAW 264.7 cells. Pretreatment with BSE improved the survival rate and suppressed alanine aminotransferase and aspartate aminotransferase expression in LPS-induced endotoxemic mice, while proinflammatory cytokines such as IL-1β, TNF-α, IL-6, cyclooxygenase-2, and iNOS decreased dramatically in the LPS-induced liver injury model via BSE treatment. Additionally, F4/80 immunostaining showed that BSE suppressed hepatic macrophage infiltration in the liver after lipopolysaccharide injection. In conclusion, BSE may be a potential nutraceutical for preventing and regulating excessive immune responses in inflammatory disease.

## 1. Introduction

In the human body, the immune system is a network of biological mechanisms that defends against pathogens, irritants, infection, injury, and toxins. Inflammation is often associated with various diseases when the body’s immune system attacks its own cells or tissues, causing inflammation and pain [1]. Plant extracts are the most abundant source of antioxidant and anti-inflammatory factors of natural products [2,3]. In particular, fruit and vegetables are rich in bioactive phytochemicals such as flavonoids, polyphenols, terpenoids, and alkaloids [4]. Generally, inflammation involves cytokine signaling through interleukin-1β (IL-1β), IL-6, and tumor necrosis factor-α (TNF-α), alongside enzymes and proteins, such as cyclooxygenase-2 (COX-2), 5-lipoxygenase (5-LOX), and matrix metalloproteinases (MMPs), which are produced and secreted by immune cells in the body [5,6].

Broccoli *(Brassica oleracea* var. italica) is rich in essential nutrients including fiber, vitamins A and C, isothiocyanates, glucosinolates, and sulforaphane, all of which affect cell development, proliferation, differentiation, and stimulate various factors related to antioxidant and anti-inflammatory responses [7]. Interest in the relationship between aging and the use of natural products as dietary supplements is growing with the increase in average life expectancy. Recently, the popularity of sprouted foods from a broad range of seeds, including broccoli, alfalfa, and red cabbage, has increased owing to their positive health effects [8,9]. For example, broccoli sprouts (BS) exert a positive influence on inflammation, oxidative stress, and cancer and they contain up to 100 times more chemoprotective compounds than mature broccoli [10,11,12]. Furthermore, sulforaphane is more abundant in BS than in other plant sprouts [13].

The biogenic precursors to sulforaphane and glucoraphanin were abundantly found in BS and were confirmed to be active in rodent models of carcinogenesis [13]. Previously, the anti-inflammatory properties of sulforaphane were investigated through the suppression of lipopolysaccharide (LPS)-stimulated COX gene expression via the regulation of various promotor elements including CCAAT-enhancer binding proteins (C/EBP), nuclear factor kappa-light-chain enhancer of activated B cells (NF-κB), and cAMP response element-binding protein (CREB) in Raw 264.7 cells [14]. Sulforaphane also activates the antioxidant defense system by regulating nuclear factor erythroid 2-related factor 2 (Nrf2)/Kelch-like ECH-associated protein 1(Keap1)-ARE Kelch-like ECH-associating protein 1-antioxidant response element signaling, which is involved in a wide range of protective functions, such as antioxidant, anti-inflammatory, anticancer, and anti-apoptotic effects [15].

LPS is a major surface membrane component in Gram-negative bacteria, exposure to which activates the innate immune response of the body in immune cells and various organs. These immune responses are involved in the secretion of proinflammatory cytokines such as IL-6, and IL-1β [16]. Researchers have used various models with LPS treatment, such as in vitro cell cultures and in vivo rodent models, to investigate anti-inflammatory agents [17,18]. For example, endotoxemia has been induced in rodents by intraperitoneal (i.p.) injection of LPS [19,20]. In response to LPS stimulation, activated immune cells produce various cytokines including TNF-α, IL-1, nitric oxide (NO), and IL-6 [21].

The objective of this study was to compare the sulforaphane content in hot water broccoli sprout extract (BSE) and ethanolic BSE using high-performance liquid chromatography (HPLC). Then, the extract with the higher sulforaphane content could be selected and the corresponding ability of this BSE to modulate the inflammatory response could be studied in LPS-stimulated RAW 264.7 macrophage cells and LPS-induced inflammatory liver injury mouse models.

## 2. Materials and Methods

### 2.1. Preparation of BSE and HPLC

BS powder was obtained from KFood. Ltd. (Gwangju, Gyeonggi-do, Republic of Korea). A total of 5 g of dry BS powder was ultrasonically extracted with 95 mL of 70% ethanol for 2 h and then filtered. After evaporation on a rotary vacuum evaporator (N-000; EYELA, Tokyo, Japan) and freeze drying, BSE was obtained. For hot water extraction, 5 g of dry BS powder was heated by refluxing with 95 mL of water, and the mixture was then cooled to room temperature, filtered, and freeze-dried. Both BSEs (ethanolic and hot water extractions) were ground to produce fine particles that were dissolved in the cell culture medium to produce 50 mg/mL BSE as stock for the experiment.

For HPLC analysis of the two BSE fractions, a water system consisting of a 996 photodiode array detector (PDA) and Phenomenex Luna C18 (ID 4.6 × 250 mm, 5 μm particle size) (Torrance, CA, USA) column was used. In addition, 0.1% formic acid distilled water (channel A) and 0.1% formic acid acetonitrile (channel B) were used as the mobile solvent system. The slope profile progressed as follows: 0–5 min, 5–20% B linear; 5–85 min, 20–30% B linear; 85–90 min, 30% B linear; 90–100 min, 30–80% B linear; 100–105 min, 80–100% B linear; and 105–110 min, 100–105% B linear. Twenty microliters of the sample was injected at 5 mg/mL and the flow rate was 1.0 mL/min. For the standard sample, sulforaphane purchased from Sigma-Aldrich (St. Louis, MO, USA), a linear range of 6.25–100 ppm sulforaphane was dissolved in methanol, and the detection wavelength was adjusted to 254 nm as the reference compound.

### 2.2. Cell Culture and Treatment

The mouse RAW 264.7 cell line was obtained from the Korean Cell Line Bank (Seoul, Republic of Korea). These macrophage cells were cultured in complete RPMI medium with 10% fetal bovine serum and 1% penicillin and streptomycin) (Nalgene Nunc International, Rochester, NY, USA) in a 5% CO_2_ incubator at 37 °C. In addition, RAW 264.7, cells were seeded in 6-well plates at a density of 5 × 10^5^ cells/well for 24 h. Cells were pretreated with BSE for 1 h; then, an inflammatory response was induced by 1 μg/mL LPS for 24 h. Finally, cells were harvested for further experiments.

### 2.3. Animals and Treatment

The experiment was performed using six-week-old male Balb/c mice (purchased from Dae Han Bio Link Co., Daejeon, Republic of Korea). Animals were maintained under constant conditions (40–60% humidity, 12 h light/dark cycle, and 20–25 °C). For animal experiments, the mice were randomly divided into four groups of six mice each: control, 20 mg/kg LPS injected, 20 mg/kg BSE (BSE dissolved in PBS) orally injected for 2 weeks before LPS injection (20 mg/kg), and Dexamethasone (Dex) administered at a dose of 5 mg/kg one day (24 h) before LPS injection. All experimental groups, except for the control group, received an LPS injection at a dose of 20 mg/kg via (i.p.) injection 2 h before euthanization for analysis using Avertin. Blood and tissue samples were collected for further experiments. All mice were treated according to the protocols reviewed and approved by the Institutional Animal Care and Use Committee of the University of Sangji, and all procedures were carried out in accordance with the approved guidelines (No. 2022–23). For survival monitoring, Balb/c mice in the corresponding groups were orally administered BSE (50 mg/kg) for 2 weeks or injected intraperitoneally with Dex (5 mg/kg) 24 h before LPS injection. On the final day, 30 mg/kg LPS dissolved in phosphate-buffered saline (PBS) was intraperitoneally injected into the mice, and survival was monitored for 84 h after LPS injection.

### 2.4. Cell Viability Assay

RAW 264.7 macrophage cells (2.6 × 10^4^ cells/well) were seeded into a 96-well plate for 24 h; these cells were incubated with 0.1, 0.5, or 1 mg/mL BSE for 24 h. Cell viability was measured using the EZ-Cytox assay kit (Wellbio, Seoul, Republic of Korea) according to the manufacturer’s instructions. Absorbance at 492 nm was measured using a BioTek Epoch Microplate reader (Winooski, VT, USA).

### 2.5. Nitrate Scavenging Ability Assay and Cytokine Release

The NO assay was performed using a NO plus detection kit (iNtRON Biotechnology, Seongnam-si, Republic of Korea) following the manufacturer’s instructions. RAW 264.7 cells (5 × 10^4^ cells) were seeded onto 96-well plates. After 24 h of culture, 1 μg/mL LPS at different concentrations (0.5 or 1 mg/mL), with or without BSE, was added to cells for cytokine detection. After 24 h of incubation, 100 μL of supernatant and an equal amount of Griess reagent were mixed, the absorbance was measured at 540 nm, and the cells were seeded in 12-well plates at a density of 2 × 10^5^ cells/well for 24 h. Cells were pretreated with BSE for 1 h and then an inflammatory response was induced with LPS (1 μg/mL) for 24 h. The supernatant of the cell culture medium was harvested for the measurement of IL-1β and TNF-α levels. An enzyme-linked immunosorbent assay (ELISA) was performed according to the manufacturer’s instructions (Invitrogen, MA, USA). To determine the effects of BSE on cytokine release in LPS-stimulated RAW 264.7 cells, the production of IL-1β and TNF-α was measured using an ELISA kit (Invitrogen, Carlsbad, CA, USA). Cells (3 Ã–10^5^) were grown in a 6-well plate for 24 h, pretreated with 0.5–1 mg/mL BSE for 1 h, and further treated with 1 μg/mL LPS with or without BSE for 24 h. The supernatants were used for ELISA analysis. ALT and AST levels are used as indicators of hepatotoxic injury. Serum ALT and AST levels were measured using a commercial assay kit (Asan Pharmaceutical Co., Seoul, Republic of Korea) according to the manufacturer’s protocol.

### 2.6. Isolation of RNA and qPCR Analysis

The total RNA of RAW 264.7 cells and live tissue was extracted using a Qiagen RNeasy Mini Kit (Cat:74106, Qiagen, The Netherlands) according to the manufacturer’s instructions. For qPCR analysis, cDNA was synthesized using RevertAid First Strand cDNA Synthesis Kit (Thermo Fisher Scientific, Waltham, MA, USA). The qPCR primers used are listed in Table 1. The QuantStudio 1 (Applied Biosystems, Foster City, CA, USA) real-time system with SYBR^®^ Green PCR master mix (Thermo Fisher Scientific, Hanover Park, IL, USA) was used for qPCR. The qPCR conditions were as follows: 94 °C for 1 min, followed by 40 cycles of 94 °C for 10 s, 57 °C for 10 s, and 72 °C for 20 s. Data were analyzed using the C_T_ method [22]. After normalization to *GADPH* levels, which are the control genes reflected in the ΔC_T_ values, the relative quantification (RQ) of the fold change for each treatment compared with that of the reference control was determined using the following equation: RQ = 2(−ΔC_T_)/2(−ΔC_T_ reference). The RQ mean and SEM fold change values were plotted.

### 2.7. Western Blot Studies

Protein expression was determined using Western blotting. RAW 264.7 cells were cultured in 90 mm cell culture dishes (1 × 10^6^ cells/dish) for 24 h. The cells were incubated with 0.5 or 1 mg/mL BSE for 1 h before LPS treatment. Next, 1 μg/mL LPS was added to the cultured cells and incubated for 24 h. Cell and liver lysates were prepared using ice-cold RIPA buffer (Thermo Fisher Scientific, Wilmington, DE, USA) containing protease inhibitors (Roche, Indianapolis, IN, USA). The BCA Protein Assay Kit (Pierce Biotechnology, Rockford, IL, USA; #23 277) was used to quantify the protein samples. Equal volumes of protein (40 µg) were loaded onto 4–20% acrylamide gels (Bio-Rad, Rockford, IL, USA). Proteins in the gel were transferred to polyvinylidene fluoride (PVDF) membranes. The membranes were then incubated with primary antibodies diluted in 1% bovine serum albumin (BSA) in TBS-Tween (TBST) buffer overnight at 4 °C. After two washes with TBST, the membrane was incubated for 1 h with secondary antibodies (anti-mouse and anti-rabbit IgG, and a horseradish peroxidase (HRP)-linked antibody, Thermo Scientific, Pittbsburgh, PA, USA). Pierce Pico ECL Western Blotting Substrate (Thermo Scientific; No. 34580) was used for visualization and band images were collected using HyBlot CL autoradiography film (Denville Scientific, Metuchen, NJ, USA; No. E3018) or the iBright™ Imaging System (Thermo Fisher Scientific, Inc., Waltham, MA, USA). *β*-actin was used as the control for normalization, and the results are listed in Table 2.

### 2.8. Histology and Immunostaining

The liver tissues were fixed with 4% paraformaldehyde overnight at 4 °C. The histological analysis and immunostaining of tissues were performed as described in our previous study [23]. Immunocytochemistry was performed using RAW 264.7 cells; 5 × 10^5^ cells were seeded to each well of a 6-well plate for 24 h, and the cells were incubated in fresh medium containing 0.1–1 mg/mL BSE extract for 24 h. Then, the cells were rinsed in PBS and fixed with 4% paraformaldehyde at room temperature (RT) for 15 min. After three washes with PBS, the cells were incubated with 0.2% Triton X-100 in PBS for 10 min, rinsed twice with PBS for 5 min, blocked with 1% BSA in PBS for 30 min at 25 °C, and incubated with ki-67 antibodies at 4 °C overnight. The samples were then washed three times for 5 min with PBS and incubated for 1 h with fluorescent secondary antibodies (Alexa Fluor 488 goat anti-rabbit IgG, diluted 1:200). After several washes, the samples were incubated with 1 μg/mL 6-diamidino-2-phenylindole (DAPI; Thermo Fisher Scientific, Waltham, MA, USA) in PBS for 3 min, and coverslips were covered with mounting solution (DAKO; Carpinteria, CA, USA; S3025). The samples were analyzed using an Olympus IX73 fluorescence microscope (Olympus Life, Tokyo, Japan). For immunohistochemistry, liver sections were deparaffinized and rehydrated using xylene and ethanol (90–100%). For antigen retrieval, samples were boiled in 10 mM sodium citrate buffer for 15 min. The tissues were blocked with buffer containing 0.01% Triton X-100 and 1% BSA for 30 min at 25 °C and incubated with F4/80 antibody for 24 h at 4 °C. The tissues were then incubated with a secondary antibody (Alexa Fluor 594 donkey anti-mouse IgG) and 1:200 BSE (diluted in PBS) for 1 h at 25 °C. The tissue was incubated with 1 μg/mL 6-diamidino-2-phenylindole (DAPI; Thermo Fisher Scientific) in PBS for 5 min, and the coverslips were covered with mounting solution (DAKO, Carpinteria, CA, USA; S3025). The samples were analyzed using a Nikon E-800 fluorescence microscope (Nikon, Tokyo, Japan) with Motic Image Advanced 3.2 software.

### 2.9. Statistical Analysis

All data in the current study are expressed as the mean ± SEM or mean ± SD of at least three independent experiments and were evaluated using one-way analysis of variance (ANOVA), followed by Tukey’s honest significant difference test using the SPSS statistical package ver. 15.0 for Windows (IBM Corp., Somers, NY, USA). Values of * *p* < 0.05, and ** *p* < 0.01 were considered statistically significant. All experiments performed with a minimum of three biological replicates involved three technical replicates.

## 3. Results

### 3.1. HPLC Analysis of Two Types of BSE

Sulforaphane, a chemical that reduces inflammation in the body, is present in high concentrations in BSE; this was used as the standard compound in the HPLC analysis of BSE in this study [24].

**Figure 1:**
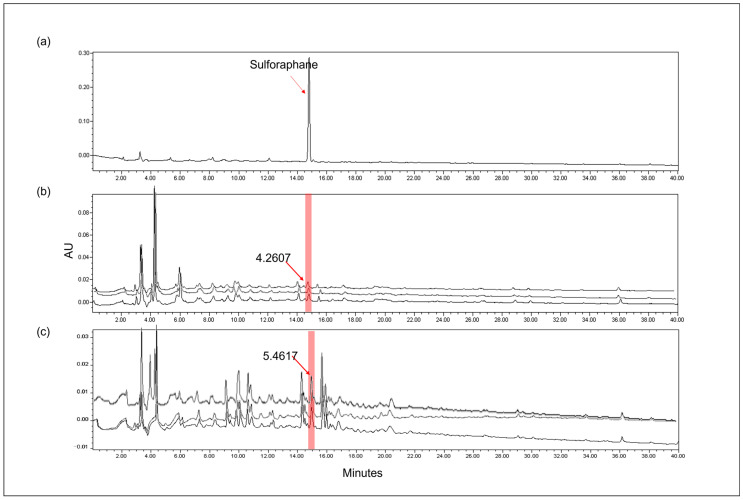
HPLC chromatogram of sulforaphane as a standard compound alongside 70% EtOH and hot water broccoli sprout (BS) extract (red area). (**a**) Sulforaphane, (**b**) hot water extract of BS, and (**c**) 70% EtOH extract of BS. The number represents mg/g of sulforaphane.

Figure 1 shows the HPLC chromatograms obtained by monitoring the detector responses at 254 nm. The retention time of the peak in sulforaphane (25 ppm) was 14.7 min (Figure 1a) and allowed an indication of the sulforaphane content in hot water and ethanolic BSE. Both BSE samples also show a peak at the same retention time as that of sulforaphane (14.7 min). The sulforaphane content differed between the water BSE and ethanolic BSE. Water BSE showed 4.2607 mg/g sulforaphane (Figure 1b), whereas ethanolic BSE showed 5.4617 mg/g sulforaphane (Figure 1c). These results indicate that ethanolic BSE contains more sulforaphane than the hot water extract (1.28-fold). Therefore, we selected ethanolic BSE for further analysis.

### 3.2. The Effect of BSE on the Cell Viability and Proliferation of RAW 264.7 Macrophages

**Figure 2:**
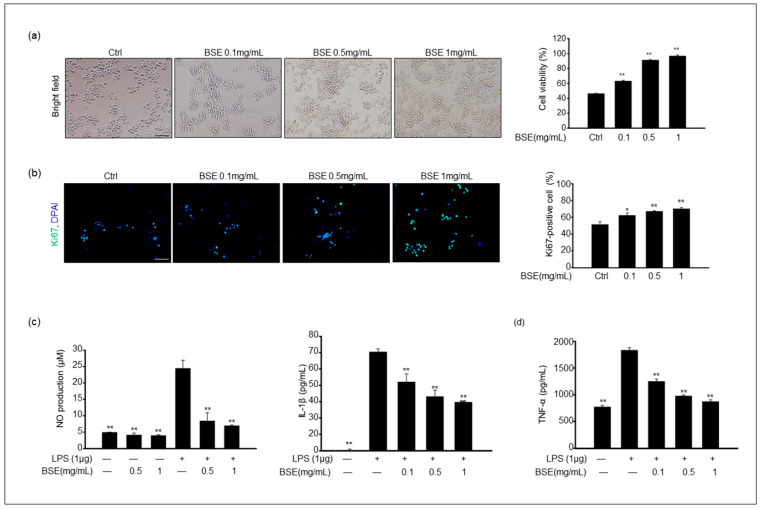
Effect of 70% EtOH broccoli sprout extract (BSE) on RAW 264.7 cell viability and production of NO, interleukin-1β (IL-1β), and tumor necrosis factor-α (TNF-α) in lipopolysaccharide (LPS)-stimulated RAW 264.7 cells. (**a**) The cell was treated with 0–1 mg/mL BSE for 24 h. The corresponding graph represents the percentage of cell viability. (**b**) BSE-treated cells were stained with Ki67 antibody, and the corresponding graph shows the percentage of Ki67-positive cells. Values of * *p* < 0.05 and ** *p* < 0.01 were considered statistically significant compared to the controls. Scale bar = 100 μm. (**c**) Cells were pretreated with BSE for 1 h and stimulated with 1 μg/mL LPS for 24 h. The levels of NO in cultured medium were quantified with Griess reagent. Values of ** *p* < 0.01 were considered statistically significant compared to cells treated with LPS only. (**d**) The levels of IL-1β and TNF-α in RAW 264.7 cells were analyzed via an ELISA. Data are shown as the mean ± standard deviation of the mean (SD) of three independent experiments. Values of ** *p* < 0.01 were considered statistically significant compared to cells treated with LPS only.

A 3-(4,5-dimethylthiazol-2-yl)-2,5-diphenyltetrazolium bromide (MTT) assay was used to measure cell viability and cytotoxicity after BSE treatment. The results showed that cell viability in the groups exposed to 0.1–1 mg/mL BSE was significantly higher than that in the control (Figure 2a). The ratio of proliferating cells was evaluated via immunostaining for Ki67 markers. The number of Ki67-positive cells was significantly increased by the BSE treatment in a dose-dependent manner compared to that in the control (Figure 2b). This result indicated that 0–0.1 mg/mL BSE was nontoxic to the RAW 264.7 cells.

### 3.3. BSE Reduces LPS-Induced NO and Proinflammatory Cytokine Production

Further analysis determined whether BSE could reduce NO production in RAW 264.7 cells in response to LPS stimulation. NO production was determined by measuring the amount of nitrite present in the cell culture medium using a NO assay kit. The cells treated with LPS alone showed a dose-dependent increase in NO production compared to the controls; this increase was dramatically attenuated in the cells that were pretreated for 1 h with 0.5–1 mg/mL BSE (Figure 2c). In addition, we measured the production of proinflammatory cytokines, such as IL-1β and TNF-α, following LPS exposure in RAW cells. IL-1β and TNF-α are strong proinflammatory cytokines that play critical roles in the immune system during inflammation [25]. The levels of both IL-1β and TNF-α were notably increased in the LPS-stimulated RAW cells, whereas pretreatment with BSE significantly suppressed the production of both IL-1β and TNF-α in a dose-dependent manner (Figure 2d).

### 3.4. BSE Inhibits the Expression of Inflammatory Genes and Proteins in LPS-Stimulated RAW 264.7 Cells

**Figure 3:**
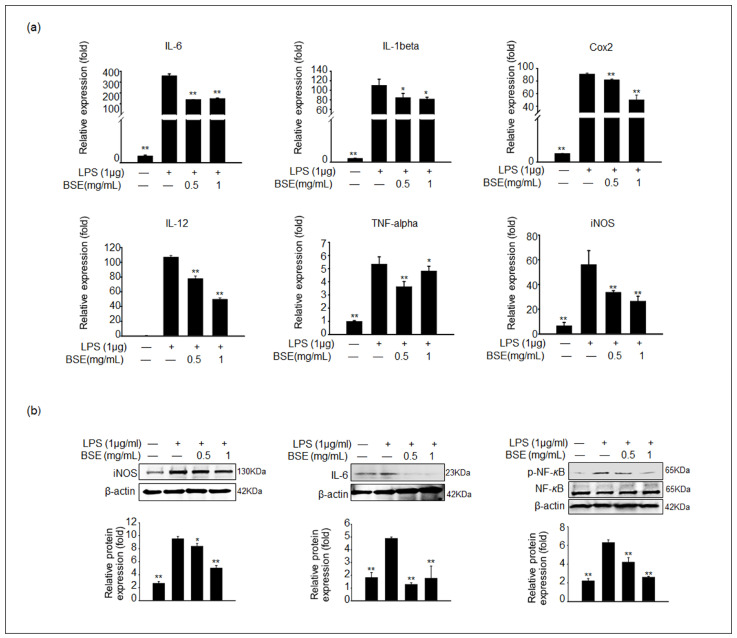
Effect of BSE on the expression of proinflammatory cytokines in LPS-stimulated RAW 264.7 cells. Cells were pretreated with BSE for 1 h before LPS treatment for 24 h. (**a**) The mRNA expression of IL-6, IL-1β, COX-2, IL-12, TNF-α, and iNOS was analyzed via qPCR (n = 5). Values represent the mean ± SEM. (**b**) The protein expression levels of iNOS, IL-6, and phosphorylated nuclear factor-κB (phospho-NF-κB) in BSE- and LPS-treated RAW cells were analyzed via immunoblotting. The density of protein bands was normalized to that of β-actin or the inactive form using Image J software Version 1.53t. Data are shown as the mean ± SD of three independent experiments. All values of * *p* < 0.05 and ** *p* < 0.01 were considered statistically significant compared to cells treated with LPS only.

Based on the results of NO and inflammatory cytokine production, the gene expression levels of proinflammatory cytokines, including *IL-6*, *IL-1β*, *IL-12*, *TNF-α*, *iNOS*, and *COX-2*, in the LPS-stimulated RAW 264.7 cells were determined via qPCR analysis. The gene expression levels of proinflammatory genes were significantly increased in the LPS-stimulated cells, and these gene expression levels were remarkably suppressed in the 0.5–1 mg/mL BSE-treated groups with LPS (Figure 3a). To verify the gene expression results, COX-2, iNOS, IL-6, and IL-1β protein levels were measured for each experimental group. The expression levels of iNOS and IL-6 proteins were consistently lower in the BSE-treated samples with LPS stimulation than in the LPS-treated samples (Figure 3b). Additionally, NF-κB is a transcription factor essential for inflammatory responses [26]. The phosphorylation of NF-κB was observed after treatment with LPS and was significantly decreased in the 1 mg/mL BSE-treated samples (Figure 3b).

### 3.5. BSE Suppressed Proinflammatory Response in an LPS-Induced Inflammation Model

**Figure 4:**
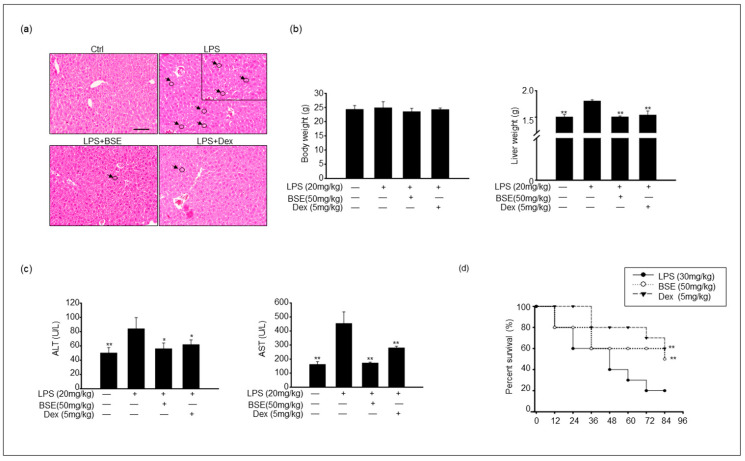
BSE ameliorated the damage and survival rate of liver tissue after an LPS challenge. (**a**) Images show hematoxylin and eosin staining of liver tissue from each experimental group: control, LPS only, BSE administration with LPS injection, and both dexamethasone (Dex)- and LPS-injected. Black arrows and circles indicate the infiltration of immune cells. Scale bar = 50 μm. (**b**) Body and liver weight from each experimental group. (**c**) Serum alanine aminotransferase (ALT) and aspartate aminotransferase (AST) levels were measured in experimental groups. (**d**) Mice were orally injected with BSE (daily) for 2 weeks. Treatment of mice with i.p. injection of Dex (5 mg/kg) 24 h before injection with 30 mg/kg LPS. On the final day, LPS was injected into the control, BSE, and Dex groups. Survival rates of mice were determined 84 h postinjection of LPS. Data are shown as the mean ± SEM of three independent experiments. Values of * *p* < 0.05, ** *p* < 0.01 were considered statistically significant compared to LPS-only groups.

A pathological analysis of the liver was performed using hematoxylin and eosin staining. There was a clear improvement in liver pathology 2 h after LPS injection. LPS exposure resulted in an increased infiltration of immune cells in the liver; however, the BSE group showed less infiltration of these immune cells in the liver tissue (Figure 4a). In addition, liver weight was significantly increased in the LPS-injected mice but not in the BSE-treated groups; nonetheless, body weight did not differ between the groups (Figure 4b). The serum concentrations of alanine aminotransferase (ALT) and aspartate aminotransferase (AST), which are released into the blood upon LPS-mediated damage to the liver tissue, were markedly lower in the BSE group (Figure 4c). Furthermore, Figure 4d shows that BSE pretreatment significantly improved the survival rate of liver cells compared with that in LPS-treated mice. The experimental group injected with LPS (30 mg/kg) showed 80% mortality 72 h after injection. Overall, 84 h after treatment, the LPS-treated group had an 80% mortality rate, the dexamethasone (Dex)-injected group had a 40% mortality rate, and the BSE (50 mg/kg) group had a 50% mortality rate (Figure 4d). As expected, pretreatment with Dex was strongly associated with reduced mortality in LPS-injected mice. The BSE-injected group also showed a significantly increased survival rate compared to the LPS-injected mice, but it was not as significant as that in the Dex group.

### 3.6. The Effect of BSE on the Gene Expression of Liver, Serum Cytokine Production, and Macrophage Infiltration in an LPS-Induced Inflammation Model

**Figure 5:**
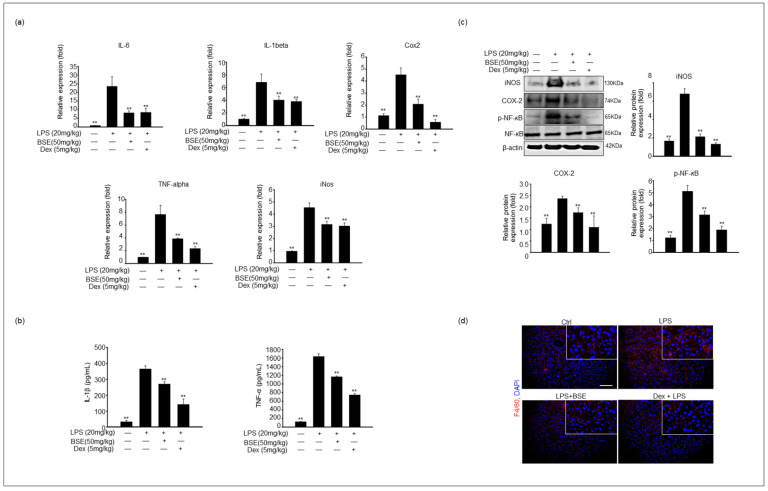
BSE suppressed the expression of proinflammatory genes and cytokine release in LPS-induced liver injury. Liver tissue samples were harvested from BSE-administrated mice for 2 weeks and then 2 h post-i.p. LPS injection (20 mg/kg). (**a**) Expression levels of *IL-6*, *IL-1β*, *COX-2*, *TNF- α*, and *iNOS* were analyzed from liver tissue of each experimental groups via qPCR. The graph shows mean ± SEM of three independent experiments. Values of ** *p* < 0.01 were considered statistically significant compared to mice treated with LPS only. (**b**) IL-1β and TNF-α levels in mice serum were analyzed with an ELISA from each experimental group. Values of ** *p* < 0.01 were considered statistically significant compared to cells treated with LPS only. Effect of BSE on the expression of inflammatory proteins and infiltration of macrophages in LPS-induced liver injury models. (**c**) The protein levels of iNOS, COX-2, and phospho-NF-κB in liver lysate were analyzed via immunoblotting. The corresponding graph shows that the density of protein bands was normalized to that of β-actin or the inactive form using Image J software Version 1.53t. Data are shown as the mean ± SEM of three independent experiments. Values of ** *p* < 0.01 were considered statistically significant compared to cells treated with LPS only. (**d**) F4/80 staining of the liver tissue shows the protein expression of hepatic F4/80 for each experimental group: control, LPS only, LPS with BSE, and Dex with LPS. The relative intensities via densitometry are presented as a graph with the mean ± SEM of three independent experiments. Scale bar = 50 μm. Values of ** *p* < 0.01 were considered statistically significant compared to mice treated with LPS only.

Based on our in vitro results, the levels of proinflammatory cytokines were assessed using qPCR and enzyme-linked immunosorbent assay (ELISA) analysis. Consistent with the results of proinflammatory gene expression in RAW cell culture by BSE, the mRNA expression levels of *IL-6*, *IL-1β*, *IL-12*, *TNF-α*, *iNOS*, and *COX-2* in liver tissue were significantly increased in the LPS-injected mice and dose-dependently decreased in the BSE-administered mice for 2 weeks and 2 h after LPS stimulation (Figure 5a). The IL-1β and TNF-α levels were measured to determine the anti-inflammatory effects of BSE in the mouse serum. The corresponding cytokine serum levels were significantly higher in the LPS-induced liver injury group than in the control group; however, when combined with BSE treatment, the cytokine levels decreased compared to those in the LPS-only group (Figure 5b). The protein expression levels in the liver were detected via immunoblotting. The corresponding iNOS and COX-2 expression levels were higher in the LPS-treated mice than in the BSE-treated groups after LPS stimulation. In addition, NF-κB was highly phosphorylated after treatment with LPS and was significantly decreased in the samples of BSE- or Dex-treated mice (Figure 5c).

Macrophages play a critical role in the pathogenesis of LPS-induced liver damage. Accordingly, we observed the infiltration of liver tissue by immune cells in an LPS-injected mouse model in this study. Next, we conducted histological studies to examine the effects of BSE on macrophage infiltration into the liver. Macrophage infiltration was measured via immunostaining with a macrophage-specific marker, the F4/80 antibody. This staining indicated that the liver tissue of the LPS-injected group possessed a significantly increased number of F4/80-positive cells; the number of F4/80-positive cells was notably decreased in the BSE + LPS experimental groups (Figure 5d). Similarly, immunoblotting results indicated a higher protein expression level of F4/80 in the liver lysate from the LPS-injected groups than in the liver of the BSE + LPS-treated groups (Figure 5d).

## 4. Discussion

The current study indicates that ethanolic BSE showed high anti-inflammatory activity and suggests possible mechanisms implemented by BSE to reduce the inflammatory response in RAW 264.7 macrophage cells and liver tissue in rodents. First, we performed a comparative analysis of hot water and 70% ethanol BSE by detecting the sulforaphane content via HPLC. These results indicate that the 70% ethanol extract contained more sulforaphane than the hot water extract, which is consistent with several prior studies. Yu et al. suggested that the highest recovery of bioactive compounds was achieved with 80% ethanol extraction because of the higher solubility of sulforaphane and phenolic compounds in ethanol than in water. Furthermore, Nga et al. determined that sulforaphane solubility in ethanol is 20 mg/mL, whereas it is 2.55 mg/mL in water [27]. In the case of cabbage, a plant similar to broccoli, the content of glucosinolates from a 70% ethanolic extract of white cabbage was approximately 5.8-fold higher than that in water [28]. Similarly, the extraction of phenolic compounds from lyophilized *Limnophila aromatica* was approximately 5-fold better in 75% ethanol and 75% methanol extracts compared to water extracts [29]. It can be postulated that there is a tendency for more effective compounds to be contained in the extracts using organic solvents such as ethanol and methanol. Our HPLC results showed a larger number of peaks in the ethanolic extract than in the hot water extract, although the components of all peaks were unknown.

According to previous studies, RAW 264.7 cells differentiate from macrophage-like cells into dendrite-like cells after LPS stimulation [30]; consequently, these dendrite-like cells activate the production of proinflammatory cytokines [31]. In our study, morphological changes in LPS-stimulated RAW 264.7, compared to the control or BSE-treated groups, were observed. In addition, BSE effectively decreased the levels of proinflammatory cytokines in LPS-stimulated RAW cells. A previous study reported that 80% methanol broccoli floret extract can inhibit NO release and NF-κB activation in LPS-stimulated RAW 264.7 cells. These results suggest that organic solvent extraction of broccoli exerts potent anti-inflammatory effects, which is similar to our findings, for which we utilized broccoli sprouts instead [32]. Generally, NO is considered a proinflammatory mediator, and its production increases in immune cells through iNOS overexpression [33]. As expected, iNOS and COX-2 expression levels were significantly elevated in LPS-stimulated RAW 264.7 cells. In our study, BSE treatment dramatically decreased iNOS and COX-2 expression. COX-2 also acts as a promising modulator of IL-β, IL-6, and TNF-α expression. Therefore, our results also demonstrate that the expression levels of IL-β, IL-6, IL-12, and TNF-α were aligned with the corresponding COX-2 levels across the experimental groups.

Several studies have indicated that BSE positively regulates various diseases. Sulforaphane-rich BS has potential anticancer properties and prevents oxidation via Nrf2 regulation [15,34]. Additionally, sulforaphane suppresses adipogenesis and lipid accumulation by regulating adipocyte differentiation [35]. Glucoraphanin, which is derived from BS, has also been reported to improve liver dysfunction in rodents. When glucoraphanin was administered (69 µmol/day) for 60 days, the plasma levels of liver function enzymes, such as ALT, AST, and γ-glutamyl transpeptidase (γ-GTP), decreased in rodents [36]. Based on these previous studies, Xu et al. described the effects of glucoraphanin on obesity-related inflammation, energy homeostasis, and insulin resistance in high-fat-diet-fed mice [37]. Similarly, our results showed that BSE significantly suppressed the LPS-induced acute inflammatory response in the livers of mice. Serum ALT and AST levels were downregulated in LPS-induced liver injury following BSE administration in mice. Many investigators have used LPS injection or a combination of LPS and D-galactosamine as an experimental model to induce liver inflammation and study the underlying molecular and cellular mechanisms of various molecules in mice. According to these results, TNF-α, IL-1β, and IL-6 were highly expressed 2 h after LPS treatment compared to 6 h; therefore, these were determined to be the most suitable markers for proinflammation in the liver after LPS treatment [38]. Based on these results, LPS was injected into mice 2 h before euthanization in the current study. As expected, these genes were highly upregulated in the LPS-induced liver injury model.

Several studies have investigated the effects of sulforaphane on liver diseases. Sulforaphane suppresses hepatic steatosis in NAFLD mice by regulating lipid metabolism disorders via upregulation of the FGF21/FGFR1 pathway [39], reduces hepatic glucose production in patients with type 2 diabetes [40], and protects the liver against traumatic hemorrhagic shock by ameliorating apoptosis and inflammation by activating the Nrf2/HO-1 pathway [41]. Ishida et al. also suggested that sulforaphane ameliorated ethanol- and carbon tetrachloride-induced liver fibrosis in mice via an Nrf2-mediated antioxidant response [42]. Similar to our results, Notarte et al. investigated that the anti-inflammatory effects of a highly selective COX-2-inhibitory flavonol-enriched butanol fraction from *Uvaria alba* (UaB) in RAW 264.7 cells, and their results showed that UaB increased cytoprotective NRF2 activity and inhibited the NF-kB pathway via the nuclear translocation of p65. In addition, according to biological significance via computation binding and reactivity experiments, UaB extract also inhibits this pathway [43].

Interestingly, in other organs, dietary sulforaphane can reverse abnormalities associated with autism spectrum disorder (ASD). For example, an experimental group of participants receiving sulforaphane showed an improvement in problems observed in ASD, such as social interaction, verbal communication, and abnormal behavior. This study suggests that sulforaphane positively affects biological changes in ASD, including oxidative stress, low glutathione synthesis, increased lipid peroxidation, and neuroinflammation [44]. According to these studies, BSE has antioxidant and anti-inflammatory effects in multiple organs.

Dex has anti-inflammatory activity, inhibits LPS-induced inflammatory responses, and is widely used to treat various inflammatory diseases [45,46]. In our study, Dex was used as a positive control to determine the anti-inflammatory effects of BSE in the livers of LPS-injected mice. The expression levels of COX-2, iNOS, and proinflammatory cytokines in the liver were reduced in BSE-administered mice with LPS injection, and the expression levels of several cytokines were similar to those in the Dex-treated groups. Therefore, these results indicate that BSE can be a powerful natural anti-inflammatory supplement. Inflammation plays a critical role in the development of chronic diseases in humans, including cancer, cardiovascular disease, atopic dermatitis, arthritis, Crohn’s disease, and diabetes [46,47]. BSE is a complex plant extract and not a single molecule, although we measured the content of major molecules (sulforaphane) in BSE using HPLC analysis. In this study, the anti-inflammatory effects of BSE were due to various bioactive substances, including sulforaphane. BSE can probably be defined more as a functional food rather than a single substance for drug treatment. Nevertheless, functional foods may provide therapeutic benefits in the prevention of various inflammation-related diseases. Therefore, the results of this study are significant. Further studies based on our results will require the isolation of various single molecules from BSE and functional tests with single molecules.

## 5. Conclusions

In conclusion, sulforaphane-rich BSE, extracted from 70% ethanol, suppressed COX-2, iNOS, and the proinflammatory cytokines IL-1β, IL-6, IL-12, and TNF-α in inflammation-inducing RAW 264.7 cells. Additionally, BSE suppressed the expression of proinflammatory cytokines and related proteins in LPS-induced liver injury. Therefore, we suggest the use of BSE from natural sources as an anti-inflammatory supplement.

## Figures and Tables

**Table 1:** List of primers for qPCR.

Gene	Forward Primer	Reverse Primer
*IL6*	5′-TGATGCTGGTGACAACCACG-3′	5′-CAGAATTGCCATTGCACAACTC-3′
*IL-1β*	5′-ACCTTCCAGGATGAGGACATGA-3	5′-CTAATGGGAACGTCACACACCA-3
*IL-12*	5′-CCAGAGACATGGAGTCATAG-3′	5′-AGATGTGAGTGGCTCAGAGT-3′
*TNF-α*	5′-CAGGCGGTGCCTATGTCTC-3′	5′-CGATCACCCCGAAGTTCAGTAG-3′
*COX-2*	5′-TCCTCACATCCCTGAGAACC-3′	5′-GAAGCCAGATGGTGGCATAC-3′
*iNOS*	5′-ACGGCAAACTGCACAAAGC-3′	5′-CGTTCTCTGAATACGGGTTGTTG-3′
*GADPH*	5′-GTCGGTGTGAACGGATTTG-3′	5′-CTTGCCGTGGGTAGAGTCAT-3′

**Table 2:** List of primary antibodies used.

Antibody	Manufacturer	Catalog Number	Dilution (Usage)
iNOS	Cell signaling	#13120	1:1000 (WB)
IL-6	Bio-rad	AAM15G	1:1000 (WB)
p-NF-κB p65	Cell signaling	#3033	1:1000 (WB)
NF-κB p65	Santa Cruz	Sc-8008	1:1000 (WB)
β-actin	Santa Cruz	Sc-47778	1:1000 (WB)
F4/80	Santa Cruz	Sc-377009	1:1000 (WB)
Ki67	Abcam	Ab15580	1:200 (IHC)
COX-2	Cell signaling	#12282	1:1000 (WB)

## Data Availability

Data is contained within the article.
